# NGF controls APP cleavage by downregulating APP phosphorylation at Thr668: relevance for Alzheimer's disease

**DOI:** 10.1111/acel.12473

**Published:** 2016-04-13

**Authors:** Viviana Triaca, Valentina Sposato, Giulia Bolasco, Maria Teresa Ciotti, Piergiuseppe Pelicci, Amalia C. Bruni, Chiara Cupidi, Raffaele Maletta, Marco Feligioni, Robert Nisticò, Nadia Canu, Pietro Calissano

**Affiliations:** ^1^ Institute of Cell Biology and NeuroscienceNational Research Council (CNR)RomeItaly; ^2^ European Brain Research Institute (EBRI Foundation)RomeItaly; ^3^ European Molecular Biology Laboratory (EMBL)MonterotondoItaly; ^4^ European Institute of Oncology (IFOM‐IEO)MilanItaly; ^5^ Regional Neurogenetic Center (CRN)ASP CatanzaroLamezia TermeItaly; ^6^ Department of System MedicineUniversity of Rome “Tor Vergata”RomeItaly

**Keywords:** AD, APP^pT668^, BACE, NGF, ShcC, TrkA–APP interaction

## Abstract

NGF has been implicated in forebrain neuroprotection from amyloidogenesis and Alzheimer's disease (AD). However, the underlying molecular mechanisms are still poorly understood. Here, we investigated the role of NGF signalling in the metabolism of amyloid precursor protein (APP) in forebrain neurons using primary cultures of septal neurons and acute septo‐hippocampal brain slices. In this study, we show that NGF controls the basal level of APP phosphorylation at Thr668 (T668) by downregulating the activity of the Ser/Thr kinase JNK(p54) through the Tyr kinase signalling adaptor SH2‐containing sequence C (ShcC). We also found that the specific NGF receptor, Tyr kinase A (TrkA), which is known to bind to APP, fails to interact with the fraction of APP molecules phosphorylated at T668 (APP^pT668^). Accordingly, the amount of TrkA bound to APP is significantly reduced in the hippocampus of *ShcC* KO mice and of patients with AD in which elevated APP^pT668^ levels are detected. NGF promotes TrkA binding to APP and APP trafficking to the Golgi, where APP–BACE interaction is hindered, finally resulting in reduced generation of sAPPβ, CTFβ and amyloid‐beta (1‐42). These results demonstrate that NGF signalling directly controls basal APP phosphorylation, subcellular localization and BACE cleavage, and pave the way for novel approaches specifically targeting ShcC signalling and/or the APP–TrkA interaction in AD therapy.

## Introduction

Accumulating evidence suggests that the NGF signalling system may be disrupted early in sporadic AD. In particular, alterations of the NGF signalling system in the basal forebrain correlate more robustly than the amyloid load with cognitive deficits in mild cognitive impairment (MCI) and with MCI progression towards AD (Mufson *et al*., 2012).

Consistent with these findings, antibody‐mediated neutralization of NGF causes increased amyloid in AD11 mice (Ruberti *et al*., 2000). Moreover, *in vitro* models of NGF deprivation show that amyloid accumulation is induced and AD pathology is mimicked in PC12 and hippocampal neurons (Matrone *et al*., 2008a,b); this has been referred to as ‘Alzheimer's‐like molecular syndrome’ (Calissano *et al*., 2010).

Furthermore, NGF treatment can ameliorate cognition and amyloidogenesis in AD transgenic models (Yang *et al*., 2014), and AD clinical trials based on NGF gene therapy have produced promising results with respect to cognitive preservation (Rafii *et al*., 2014).

Despite these findings, the molecular mechanisms underlying the neuroprotective action of NGF in AD‐like neurodegeneration remain unclear. In particular, elucidating how NGF modulates APP processing in neurons is critical for our basic understanding of the phenomenon and for the early diagnosis and treatment of AD.

Amyloid precursor protein (APP) can be processed via alternative physiological or amyloidogenic pathways depending on whether cleavage occurs via alpha secretase 10/17 (ADAM 10/17) or beta secretase 1 (BACE1) in neurons, which causes the generation of alpha (sAPPα, CTFα) or beta (sAPPβ, CTFβ) products, respectively (Bernstein *et al*., 2009; Vassar *et al*., 2014).

APP phosphorylation pattern has been reported to be crucial for APP binding to cytosolic interactors and for its subcellular trafficking and final processing (Suzuki & Nakaya, 2008; Tamayev *et al*., 2009). Particularly, phosphorylation of APP at T668 (APP695 isoform numbering) increases the beta product levels by facilitating exposure to and cleavage by BACE in human patients with AD and transgenic models (Lee *et al*., 2003; Shin *et al*., 2007). The APP^pT668^ form is neuron‐specific, highly expressed in AD dystrophic neurites and amyloid plaques, and induces neurodegeneration (Lee *et al*., 2003; Chang *et al*., 2006). Of interest, mice bearing a Thr to Ala substitution at 668 (T668A) prevent AD cognitive deficits when crossed to the FDD_KI_ mouse model of dementia (Lombino *et al*., 2013). Accordingly, the inhibition/reduction of T668 phosphorylation may be a potential target for AD therapy (Chang *et al*., 2006).

Despite many studies on the anti‐amyloidogenic action of NGF in the brain, to date the effect of NGF on APP^T668^ phosphorylation has not yet been investigated. Thus, we investigated the NGF‐mediated control of APP phosphorylation/processing in the forebrain and the underlying molecular mechanisms using *in vitro* (primary septal neurons), *ex vivo* (septo‐hippocampal [SH] slices), and *in vivo* (KO mice and AD brain tissues) experimental paradigms.

Our results show that NGF rescues neurons from JNK(p54)‐mediated phosphorylation of APP through its early‐downstream signalling adaptor ShcC. The reduction in APP phosphorylation induced by NGF favours APP binding to its receptor TrkA, while affecting APP interaction with BACE, and reduces the generation of beta products, such as CTFβ and amyloid (1‐42). These findings suggest that NGF control of APP phosphorylation and the subsequent events are potentially disrupted in the human AD brain where the APP^pT668^ level is elevated and the APP–TrkA interaction is dramatically reduced.

Overall, our data support a role of APP (T668) phosphorylation in the NGF‐driven control of amyloidogenesis in the basal forebrain and suggest early‐downstream effectors of the NGF pathway as targets in the development of novel anti‐amyloidogenic therapies for AD.

## Results

### NGF controls basal APP phosphorylation at T668

The phosphorylation of APP at T668 is known to facilitate APP amyloidogenic processing and AD pathology (Lee *et al*., 2003; Chang *et al*., 2006).

**Figure 1:**
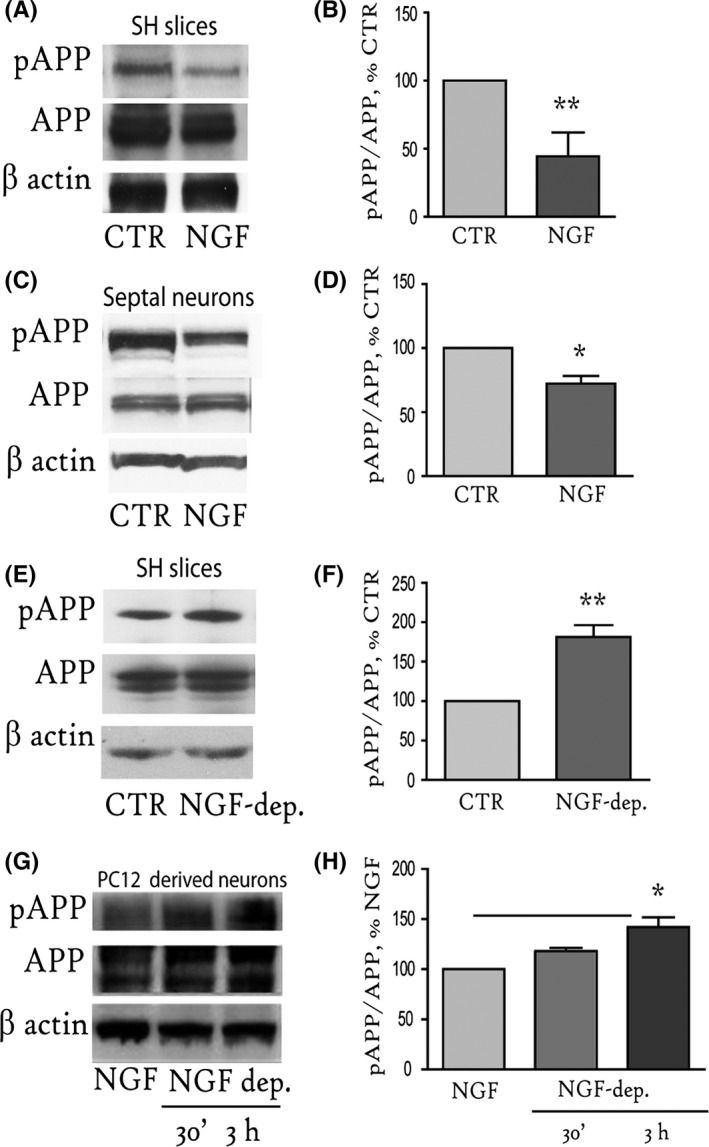
NGF controls basal APP phosphorylation at T668 in neurons. (A, C). Representative Western blots (WB) of full‐length APP^pT668^ and total APP on extracts from NGF‐treated (100 ng mL^−1^, 1 h) and control (CTR) septo‐hippocampal slices (A) or mouse septal neurons (C) are shown. (B, D). The results are reported as percentage of control SH slices (44.4 ± 17.5% of CTR, **P < 0.01) or septal neurons (72.3 ± 6.1% of CTR, *P < 0.05). (E) Extracts from SH slices incubated with anti‐NGF neutralizing antibodies (NGF‐dep; 6 h) were immunoblotted with anti‐phosphorylated and total APP. (F) Bar graphs depict quantification of the immunoreactivity for the APP^pT668^ band normalized to the total APP level (195.9 ± 5.3% of CTR, *P < 0.05). (G) PC12 cells were cultured with NGF (100 ng mL^−1^) for 10 days (CTR) and then deprived of NGF for 30′ or 3 h (NGF‐dep 30′ and NGF‐dep 3 h, respectively). (H) Bar graphs depict quantification of the immunoreactivity for the APP^pT668^ band normalized to the total APP level (142.1 ± 9.9% of CTR, *P < 0.05). Beta‐actin was used as a loading control. Triplicate data from 4 to 5 independent experiments were used for the analysis. SH = septo‐hippocampal.

To assess the effect of NGF on APP phosphorylation at T668 in the forebrain, SH slices from control mice were incubated with NGF (100 ng mL^−1^; 1 h) and analysed by Western blot (WB) using specific antibodies against APP^pT668^ and total APP (Fig. 1A). The APP^pT668^ level was significantly reduced in the NGF‐treated SH slices compared with the untreated slices (CTR, 44.4 ± 17.5%, *P < 0.05, Fig. 1B). Next, we restricted our analysis to the septal neurons (DIV10, E18), which are TrkA‐expressing neurons and the primary cellular target of NGF in the SH system. We incubated mouse septal neurons with NGF (100 ng mL^−1^; 1 h) and confirmed that the APP^pT668^level (Fig. 1C) was significantly decreased after NGF treatment (72.3 ± 6.1% of CTR, *P < 0.05; Fig. 1D), indicating that the effect of NGF on APP^pT668^ is specifically directed towards these neurons. The NGF‐driven decrease in APP^pT668^ does not result from APP protein degradation because the total amount of APP protein was unaffected by NGF in both paradigms (Fig. 1A,C). The same experiment was performed using rat primary neurons with comparable results (Fig. S1A,B).

We next assessed whether in turn NGF withdrawal promotes APP phosphorylation by incubating SH slices with anti‐NGF neutralizing antibody. We analysed APP^pT668^ and total APP by WB (Fig. 1E) and found almost doubled levels of APP phosphorylation following 6‐h incubation with anti‐NGF antibody (195.9 ± 5.3% CTR, **P < 0.01; Fig. 1F).

We also used the classic NGF deprivation paradigm in the pheochromocytoma cell line (PC12). PC12 cells were differentiated into neurons by culturing in NGF (100 ng mL^−1^) under serum‐free conditions for 10 days. Then, the PC12‐derived neurons were switched to both serum‐free and NGF‐free media for the indicated time periods (30′, 3 h) and analysed by WB for APP^pT668^ and total APP (Fig. 1G). As shown in Fig. 1H, the APP^pT668^ level progressively increased after 30′ of NGF withdrawal and reached a significantly higher level after 3 h of NGF withdrawal (142.1 ± 9.9% of CTR, *P < 0.05). The total APP level did not change after 30′ or 3 h of NGF deprivation.

Our data indicate that NGF controls basal APP phosphorylation at T668 in septal neurons *in vitro* and *ex vivo*.

### NGF treatment decreases activation of JNK (p54) kinase

**Figure 2:**
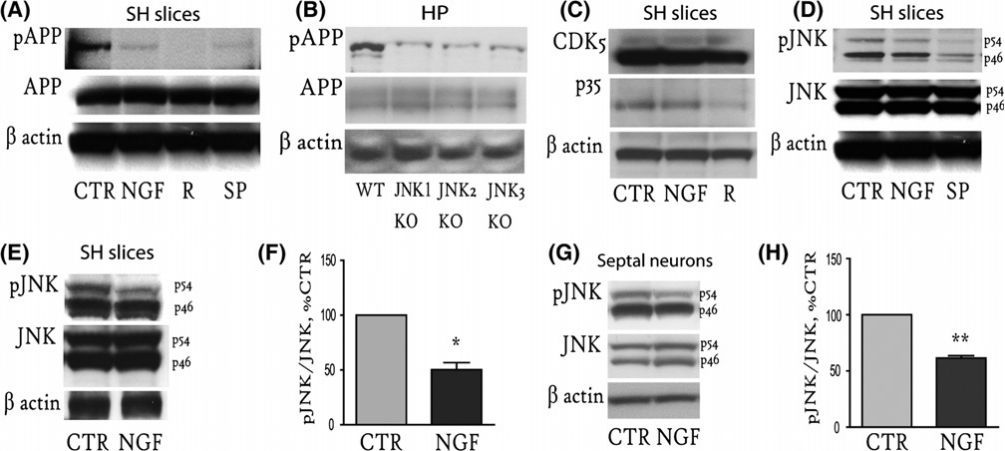
NGF treatment causes decreased activation of the JNK (p54) kinase. (A). Representative WB against APP^pT668^ and total APP of extracts from septo‐hippocampal slices untreated (CTR) or treated with NGF (100 ng mL^−1^, 1 h), or SP600125 (SP; 2 μm, 1 h), or roscovitine (R, 14 μm, 1 h). (B) Representative WB against full‐length APP^pT668^ and total APP from hippocampal extracts of *JNK*_*1*_, *JNK*_*2*_ and *JNK*_*3*_ KO mice. (C) Extracts from control SH slices (CTR) or SH slices treated with NGF (NGF; 100 ng mL^−1^, 1 h) or roscovitine (R; 14 μm, 1 h) were blotted against CDK5 and its regulatory subunit p35. (D) Representative WB of extracts from control SH slices (CTR) or SH slices treated with NGF (100 ng mL^−1^, 1 h) or SP600125 (SP; 2 μm, 1 h) using antibodies against pJNK and total JNK. Two bands of 46 and 54 kDa were detected by WB. (E, G). Representative WB using antibodies against pJNK and total JNK of extracts from untreated (CTR) and NGF‐treated (100 ng mL^−1^, 1 h) SH slices (E) or mouse septal neurons (G). (F, H) The levels of phosphorylated p54 in SH slices (F) or mouse septal neurons (H) were measured, and the ratio was calculated over the total p54 level, and expressed as percentage of the control (59.4 ± 4.4% of CTR, *P < 0.05 and 61.5 ± 2.1% of CTR, **P < 0.01, respectively). Beta‐actin was used as a loading control. Triplicate data from 4 to 5 independent experiments were used for the analysis. SH = septo‐hippocampal, HP = hippocampus.

Given that JNK (Standen *et al*., 2001; Colombo *et al*., 2009; Sclip *et al*., 2011) and cyclin‐dependent kinase 5 (CDK5) (Iijima *et al*., 2000; Liu *et al*., 2003) are Ser/Thr kinases and regulate APP phosphorylation under basal and pathological conditions, they were analysed as potential mediators of the NGF effect on APP^pT668^. We incubated SH slices with the CDK5 and JNK inhibitors roscovitine (R; 14 μm, 1 h) and SP600125 (SP; 2 μm, 1 h), respectively and found a dramatic reduction in the APP^pT668^ level (Fig. 2A). The inhibitor concentrations and incubation times were selected to produce the maximum inhibitory effect on the specific kinase without affecting cell survival (data not shown). We analysed APP phosphorylation levels also in mouse models of genetic ablation of the three JNK isoforms (JNK1‐3) and found reduced APP^pT668^ levels in the hippocampal extracts of JNK1, JNK2 and JNK3 KO mice (*n* = 2 mice per genotype) (Fig. 2B).

We observed that NGF does not affect the level and/or activation of CDK5. Briefly, SH slices were incubated with NGF (100 ng mL^−1^, 1 h) or roscovitine (R; 14 μm, 1 h), and WB was performed using antibodies against CDK5 or p35, the CDK5 activator (Fig. 2C). NGF was not able to modulate CDK5 or p35 levels, while the p35 level was affected by roscovitine, as expected (Fig. 2C). SH slices were then incubated with NGF (100 ng mL^−1^, 1 h) or SP600125 (SP; 2 μm, 1 h), and JNK activation was analysed by WB. A decrease in the phosphorylation of the two JNK bands of 46 (p46) and 54 (p54) kDa was observed upon SP treatment, while NGF specifically affected the activation of only the p54 isoform (Fig. 2D).

Therefore, we repeated the analysis to quantify the phosphorylated p54 level in the SH slices treated with NGF (100 ng mL^−1^, 1 h) and observed a significant reduction in the phosphorylation level of the JNK(p54) isoform compared with the untreated slices (59.4 ± 4.4% of CTR, *P < 0.05; Fig. 2E,F). A similar correlation was also observed in mouse septal primary neurons, in which we observed a significant decrease in JNK(p54) phosphorylation upon NGF treatment (61.5 ± 2.1% of CTR, **P < 0.01; Fig. 2G,H). Given that the downregulation of p54 phosphorylation observed with NGF treatment was comparable to that observed using SP600125 (Fig. 2D) and that both types of treatment decreased the APP^pT668^ level (Fig. 2A), our results suggest that NGF acts through pJNK(p54) to decrease APP^pT668^. When treating with NGF rat primary neurons, we obtained comparable results (Fig. S1A,B).

Thus, our results correlate the effect of NGF on the APP^pT668^ level with a specific reduction in JNK(p54) activation and indicate JNKs in the control of APP phosphorylation in the cholinergic forebrain.

### Deletion of the ShcC gene causes increased pJNK activation and APP^pT668^

**Figure 3:**
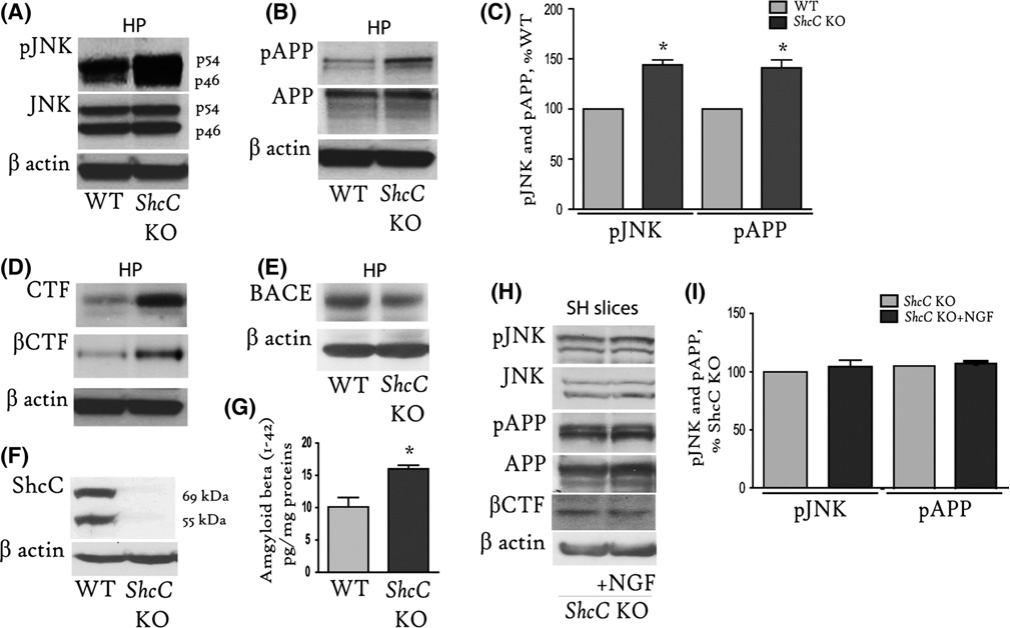
Deletion of the ShcC gene results in increased levels of pJNK, APP^pT668^, sAPPβ, CTFβ and amyloid‐beta (1‐42). (A, B) Representative WB of hippocampal extracts from *ShcC* KO (*n* = 3) and WT (*n* = 3) mice using antibodies against pJNK and total JNK (A), or APP^pT668^ and C‐terminal APP (B). (C) The graph illustrates the optical density analysis of pJNK (144 ± 5% of WT, *P < 0.05) and APP^pT668^ (141 ± 8% of WT, *P < 0.05) measured over total JNK and APP in *ShcC* KO mice, and the values are reported as a percentage of the values in WT mice. (D–F) A representative WB of hippocampal extracts from *ShcC* KO and age‐matched WT mice probed with specific antibodies against APP (APP C‐ter and 6E10; D), BACE (E) and ShcC (F). (G) Endogenous amyloid‐beta (1‐42) levels in hippocampal extracts of *ShcC* KO (16 ± 0.6 pg mg^−1^ proteins) and age‐matched wild‐type mice (10 ± 1.4 pg mg^−1^ proteins, *P < 0.05). *N* = 3 mice per genotype were used for this analysis. (H, I) SH slices from *ShcC* KO mice were incubated with NGF (100 ng mL^−1^, 1 h), extracted and probed for phosphorylated and total APP and JNK, and the CTFβ (H). The data are reported as percentage of control in the graph in (I). Levels of pJNK (102 ± 1.1% of *ShcC* KO; *P* = 0.096) and APP^pT668^ (104.5 ± 5.7% of *ShcC* KO; *P* = 0.37) are unchanged following NGF incubation. Beta‐actin was used as a loading control. Data from two separate experiments with brain slices from four animals per genotype were used for the analysis. HP = hippocampus; WT = wild‐type; SH = septo‐hippocampal.

Isoform C of the SH2‐containing sequence adaptor (ShcC) couples NGF/TrkA activation with neuronal inhibition of JNK (Molton *et al*., 2003). We thus hypothesized that ShcC signalling could act early downstream of NGF to downregulate pJNK and, consequently, APP^pT668^. To investigate this potential mechanism, we examined pJNK (Fig. 3A) expression in hippocampal tissue from *ShcC* KO mice by WB and found an almost 50% increase in the pJNK level compared with the age‐matched WT mice (144 ± 5% of WT, *P < 0.05; Fig. 3C). Consistent with our hypothesis, the basal APP^pT668^ level determined by WB (Fig. 3B) was found to be almost 50% increased (141 ± 8% of WT, *P < 0.05, Fig. 3C). Moreover, elevated amounts of the APP C‐terminal fragment (CTF), and in particular CTFβ (as indicated by the anti‐APP_6E10_ antibody; Fig. 3D), in the presence of normal BACE levels (Fig. 3E) were observed in the *ShcC* KO mice. The ablation of the gene product was verified probing ShcC KO and WT tissues with specific antibodies against ShcC (Fig 3F). The beta‐amyloid (1‐42) levels, measured by a highly sensitive rodent‐specific ELISA, increased in the hippocampus of *ShcC* KO (16 ± 0.6 pg mg^−1^ proteins), as compared to age‐matched control mice (10 ± 1.4 pg mg^−1^ proteins, *P < 0.05; Fig. 3G).

Furthermore, NGF stimulation (100 ng mL^−1^, 1 h, RT) of SH slices from *ShcC* KO mice (*ShcC*KO+NGF) failed to reduce JNK activation (102 ± 1.1% of *ShcC* KO, *P* = 0.096) and APP phosphorylation (104.5 ± 5.7% of *ShcC* KO, *P* = 0.37), as shown in Fig. 3H‐I. Also, levels of CTFβ are comparable between NGF‐treated and untreated SH slices from *ShcC* KO (Fig. 3H).

Our results indicate that ShcC deletion prevents NGF from its basal regulation of pJNK and APP^pT668^ and suggest that releasing the NGF‐TrkA‐ShcC control of APP metabolism leads to increased CTFβ and amyloid (1‐42) generation *in vivo*.

### APP does not interact nor colocalize with TrkA, upon phosphorylation at T668

**Figure 4:**
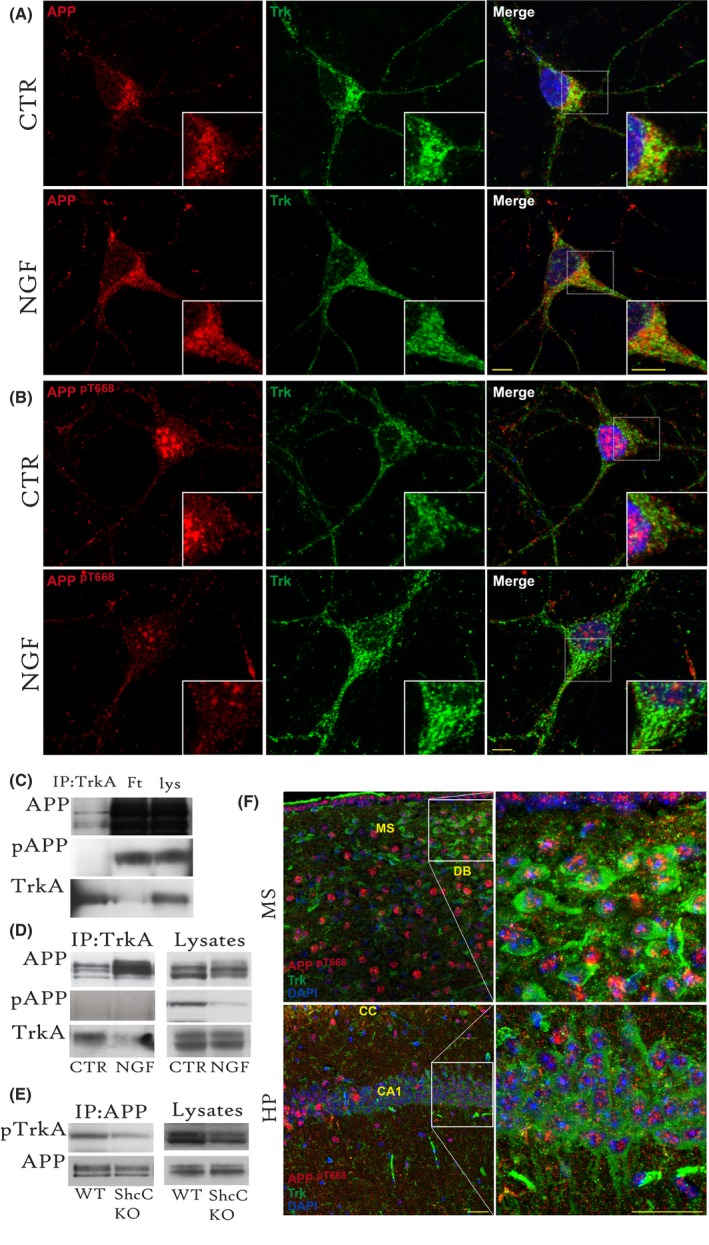
TrkA colocalizes with and binds to APP but not APP^pT668^. (A, B). Confocal microscopy images of untreated (CTR) and NGF‐treated (100 ng mL^−1^, 1 h) septal neurons are shown. Double immunofluorescence staining was performed with (A) anti‐APP C‐ter (Y188; *red*) and anti‐Trk (*green*) or with (B) specific anti‐APP^pT668^ (*red*) and anti‐Trk antibodies (*green*). Representative images are from 50 neurons per experimental group. Scale bar: 5 μm. (C, D). IP with a specific anti‐TrkA antibody was performed on extracts from control mouse hippocampal tissue (C) or untreated (CTR) and NGF‐treated (NGF; 100 ng mL^−1^, 1 h) SH slices from C57/Bl6 mice (D). Immunoprecipitates (IPs) were then probed using WB with antibodies against APP (22c11) or APP^pT668^. Triplicate data from hippocampal tissues and SH slices of at least 4–5 control C57/Bl6 mice per experimental group were used for the analysis. (E). IP with the anti‐APP C‐terminal antibody was conducted on hippocampal extracts from *ShcC* KO mice (KO; *n* = 4) and age‐matched control mice (WT; *n* = 4). IPs were probed with anti‐pTrkA and with anti‐APP (22c11) antibodies. (F). Confocal microscopy images of medial septum (MS) and hippocampal CA1 (CA1) in coronal brain sections of *ShcC* KO mice (*n* = 3 mice). Double immunofluorescence staining was performed with specific anti‐APP^pT668^ (*red*) and anti‐Trk antibodies (*green*). Scale bars: 25 μm. IPs, immunoprecipitate; Ft, flowthrough; Lys, lysate; WT, wild‐type; MS, medial septum; DB, diagonal band of Broca; HP, hippocampus; CC, Corpus Callosum; CA, Cornus Ammonis.

TrkA receptor has been shown to interact with APP *in vitro* and *in vivo* (Tarr *et al*., 2002; Matrone *et al*., 2011). Because APP undergoes a conformational change (Ramelot & Nicholson, 2001) upon T668 phosphorylation that detaches its cytoplasmic interactors (Ando *et al*., 2001), we investigated whether T688 phosphorylation affects TrkA binding to APP. We analysed the intracellular localization of Trk, total APP and APP^pT668^ using confocal microscopy. Septal neurons were treated with NGF (NGF; 100 ng mL^−1^, 1 h) and assayed by double immunofluorescence staining for Trk and APP (Fig. 4A and at low magnification in Fig. S2A), or Trk and APP^pT668^ (Fig. 4B and at low magnification in Fig. S2B). Weak APP/Trk colocalization can be detected at the plasma membrane and in the dendrites of septal neurons (CTR; *r* = 0.38 ± 0.03). Upon NGF stimulation (NGF, Fig. 4A; *r* = 0.67 ± 0.08), a stronger and statistically significant colocalization signal (*P* < 0.05) was observed, mainly in the perinuclear area (inset of Fig. 4A). Conversely, Trk and APP^pT668^ failed to colocalize in both the untreated (CTR; *r* = 0.15 ± 0.03) and NGF‐stimulated (NGF; *r* = 0.12 ± 0.03) neurons (Fig. 4B) even though the APP^pT668^ staining was present and diffuse in both the cell soma and dendrites. No statistical significance in pixel colocalization of Trk and APP^pT668^ was found in the NGF‐treated and untreated neurons (Costes threshold, *P* < 0.5; (Costes *et al*., 2004)).

Moreover, to investigate whether TrkA and APP^pT668^ interact, we resorted to immunoprecipitation (IP) analyses. Lysates of hippocampal tissues from C57/Bl6J mice were subjected to IP with a specific anti‐TrkA antibody, and then probed with anti‐APP^pT668^ or total APP (22c11) and trk antibodies. We confirmed that APP co‐immunoprecipitates with TrkA, as expected (Fig. 4C). In contrast, under the same experimental conditions, APP^pT668^ failed to co‐immunoprecipitate with TrkA (Fig. 4C). The specific interaction of TrkA with APP and not APP^pT668^ was further confirmed by IP studies with SH slices. The lack of an APP^pT668^–TrkA association was also observed in both the untreated and NGF‐treated SH slices (Fig. 4D) even though the amount of total APP that immunoprecipitated with TrkA increased upon NGF treatment (Fig. 4D).

Our data indicate that NGF stimulation promotes APP binding to its receptor TrkA but that only the fraction of APP that is not phosphorylated at T688 is able to interact with TrkA. Therefore, it was not surprising to observe that TrkA IP by APP was almost completely lost in the *ShcC* KO mice (Fig. 4E), a mouse model with elevated APP^pT668^ (Fig. 3B) Furthermore, we investigated the Tyr 490‐phosphorylated form of TrkA (pTrkA) because of the selective NGF‐driven docking of Shc to pTrkA. The levels of APP (Fig. 3B,D; Fig. 4E) and pTrkA (Fig. 4E) in the *ShcC* KO mice were found to be unaffected. The weak residual APP–TrkA binding observed in the *ShcC* KO mice may result from the presence of other Shc isoforms in the adult brain, presumably ShcB (Liu & Meakin, 2002).

We also studied APP^pT668^ and Trk localization in the absence of ShcC by double IF of coronal brain sections from perfused *ShcC* KO (Fig. 4F) and WT age‐matched mice (not shown). In particular, the cholinergic cell soma in the medial septum (MS) and diagonal band of Broca and their terminals innervating the CA1 region of the hippocampus (HP) were analysed in *ShcC* KO. The analysis confirmed *in vitro* confocal imaging and showed that Trk and APP^pT668^ do not colocalize *in vivo* in *ShcC* KO mice, neither in the MS (*r* = 0.03 ± 0.01) nor in the CA1 (*r* = 0.08 ± 0.01).

These data indicate that TrkA will colocalize and interact with APP as long as none of it is phosphorylated at T668. Accordingly, the APP–TrkA interaction is significantly affected in the *ShcC* KO brain, where high levels of APP phosphorylation occur.

### The APP–TrkA interaction is significantly affected in the hippocampus of AD patients

**Figure 5:**
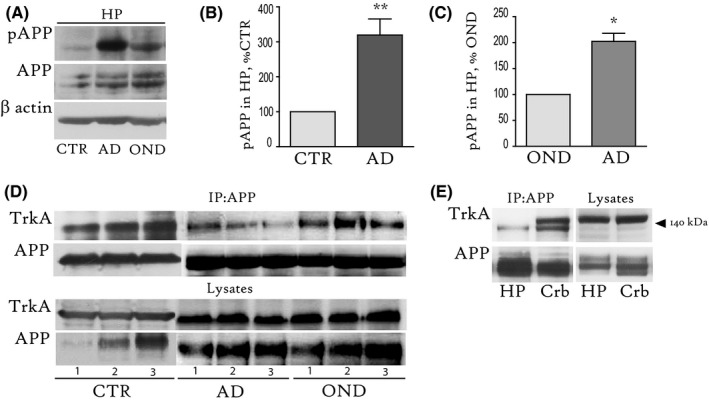
APP^pT668^ is elevated and conversely APP‐TrkA binding is reduced in the AD human hippocampus. (A). A representative WB with specific antibodies against APP^pT668^ and total APP on human hippocampal extracts from control (CTR), AD and other neurological disease (OND). (B, C) The graph shows the quantification of the APP^pT668^ level, reported as percentage of the level observed in the CTR (319.5 ± 46.1% of CTR, **P < 0.01; B) or OND group (202.4 ± 15.6% of OND, *P < 0.05; C). (D). IP with anti‐APP C‐terminal antibody was conducted on hippocampal extracts from CTR, OND and AD hippocampi. IPs were probed using WB with specific anti‐TrkA and anti‐APP N‐Ter (22c11) antibodies. (E). IP with anti‐APP C‐terminal antibody was conducted on hippocampal and cerebellar extracts from the AD_2_ subject. IPs were probed using WB with a specific antibody against TrkA and the anti‐APP (22c11) antibody. β‐actin was used as a loading control. CTR_1‐3_ = Subjects without neurological diseases. Mean age at death: 60 ± 7 years. OND_1_ = Huntington's Disease (HD); OND_2_ = spinocerebellar ataxia 17 (SCA17); OND_3_ = counter‐lateral hemisphere of unilateral intracerebral bleeding. AD_1‐3_ = familial AD (Met146Leu mutation, PS1 gene), Clinical Dementia Rating (CDR) stage 5, Braak stage VI. Mean age at death: 48 ± 8.2 years. IPs = immunoprecipitates; HP = hippocampus; Crb = cerebellum; CTR, age‐matched controls without neurological diseases; AD = Alzheimer's disease; OND = other neurological disease.

We hypothesized that the APP–TrkA interaction is affected in the AD brain, where an elevated APP^pT668^ level is often reported (Lee *et al*., 2003; Chang *et al*., 2006). To test this hypothesis, we analysed the APP^pT668^ level using WB (Fig. 5A–C) and the APP–TrkA interaction using IP (Fig. 5D,E) in brain samples from control subjects without neurological diseases (CTR; *n* = 3), patients with AD (AD; *n* = 3) and subjects affected by other neurological diseases (OND; *n* = 3). We observed a dramatic increase in the APP^pT668^ level in the hippocampus of patients with AD (Fig. 5A), as compared to both CTR (319.5 ± 46.1% of CTR, **P < 0.01; Fig. 5B) and OND (202.4 ± 15.6% of OND, *P < 0.05; Fig. 5C), as expected. Then, we immunoprecipitated the APP from the CTR, OND and AD hippocampal tissue and probed the samples with a specific anti‐TrkA antibody. We found that the amount of TrkA bound to APP was significantly lower in all three AD samples, as shown in Fig. 5D. Analysis of the TrkA‐positive co‐immunoprecipitated band relative to the input showed lower binding in AD (*P < 0.05; 56.4 ± 12, %CTR), as compared to both CTR and OND (108 + 15, %CTR), while no difference was found between CTR and OND (*P* = 0.6) hippocampal samples.

Next, we repeated the IP analysis using the hippocampal and cerebellar extracts from the same AD subject (AD2) and found that the APP–TrkA interaction was almost completely undetectable in the hippocampus of this AD patient but preserved in the AD cerebellum (Fig. 5E).

Our data reveal that a lack of APP–TrkA binding correlates with an elevated APP^pT668^ level and it is specific of neurodegenerating tissues in humans with AD.

### NGF promotes the APP binding with TrkA versus BACE1, by inducing APP trafficking to the Golgi, and reduces beta products generation

**Figure 6:**
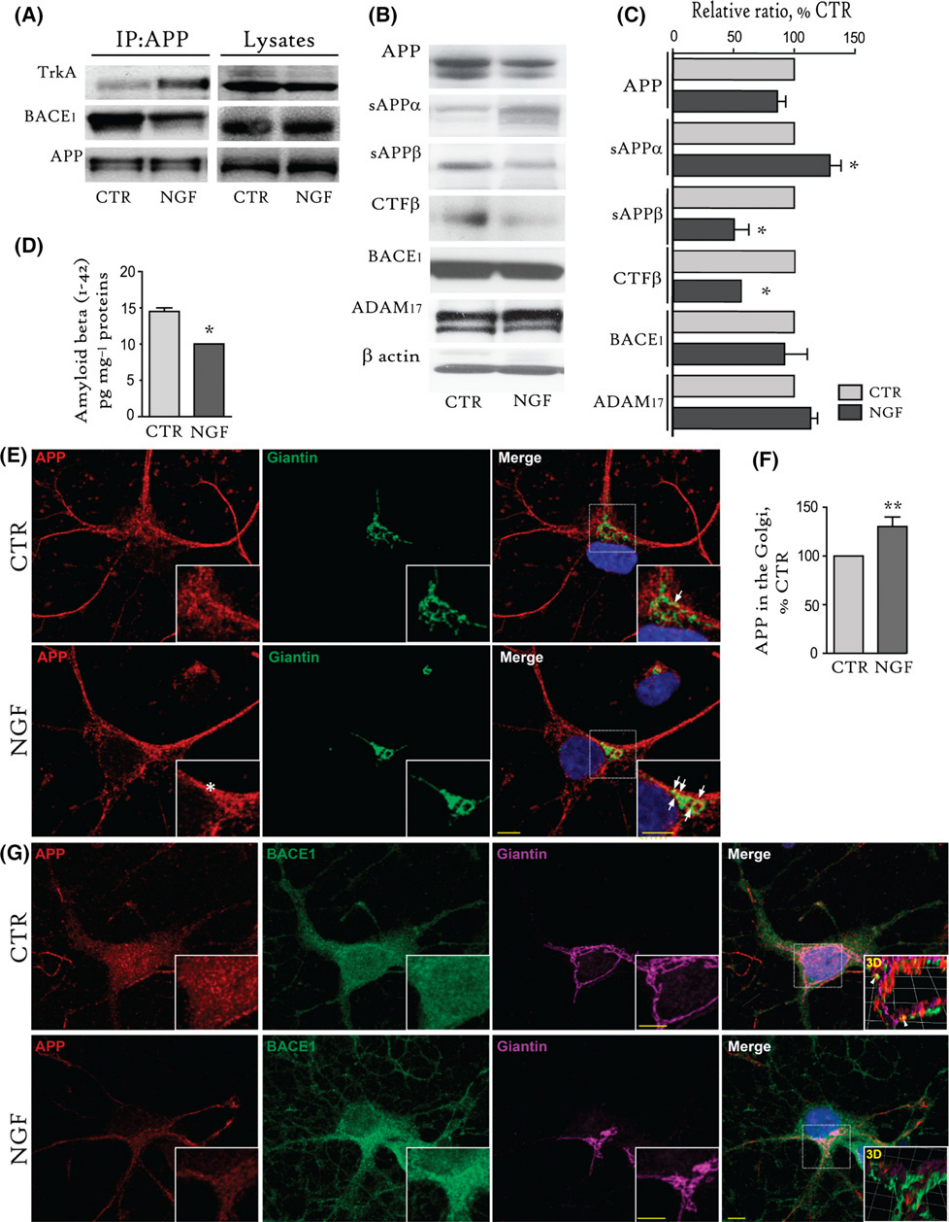
NGF favoured APP binding to TrkA instead of BACE1 and induced APP trafficking to the Golgi compartments. (A) IP with an anti‐APP C‐Ter antibody was conducted on extracts from untreated (CTR) and NGF‐treated (NGF; 100 ng mL^−1^) SH slices. IPs were probed with antibodies against TrkA, BACE1 and APP (22c11). (B, C). A representative WB (B) and statistical analysis (C) of extracts from untreated (CTR) or NGF‐treated (NGF; 100 ng mL^−1^, 1 h) SH slices for APP (86.2 ± 6.9% of CTR, *P* = 0.12), sAPPα (129.4 ± 9.3% of CTR, *P < 0.05), sAPPβ (50.6 ± 12.2% of CTR, *P < 0.05), CTFβ (52.5 ± 7.3% of CTR; *P* > 0.05), BACE1 (92.2 ± 18.7% of CTR, *P* = 0.69), ADAM17 (113.6 ± 5.8% of CTR, *P* = 0.08), and β‐actin as a loading control, are shown. (D) Endogenous amyloid‐beta (1‐42) levels (pg mg^−1^ proteins) in control (CTR; 14.5 + 0.5 pg mg^−1^ proteins) and NGF‐treated (NGF; 10 + 0.1 pg mg^−1^ proteins, *P < 0.05) SH slices. (E–G). Confocal image analysis of untreated (CTR) and NGF‐treated (NGF; 100 ng mL^−1^; 1 h) septal neurons. (E) Double immunostaining for APP/giantin in septal neurons is shown. (F) The quantitative analysis of the APP (*red*) staining in the Golgi network (anti‐giantin, *green*) upon NGF treatment by 3D quantification of the APP‐positive voxels within the giantin‐positive volume (Golgi, arrows) was performed by the Imaris 7.6 software. The results are reported in the graph as a percentage of the value in untreated neurons (130 ± 9.7% of CTR, **P < 0.01). Note the increase in membrane localization of APP in septal neurons upon NGF stimulation (E, asterisks). (G) Triple immunostaining for APP (*red*)/BACE_1_ (*green*)/giantin (*purple*) in septal neurons is shown. Note that APP immunoreactivity is enriched in the cell soma and accumulates in the Golgi upon NGF treatment, while BACE_1_ localization is more diffuse and distributed throughout the cytoplasm of both unstimulated (CTR) and NGF‐treated (NGF) septal neurons. APP colocalization with BACE was measured by creating a 3D mask of the giantin‐positive volume (inset 3D), with the help of Volocity 6.0 software, and was found to be significantly reduced upon NGF treatment (*P* < 0.05; *r* = 0.323 ± 0.049), as compared to CTR neurons (*r* = 0.579 ± 0.084). Inset 3D shows rendering of APP (*red*) and BACE (*green*)‐positive voxels within the giantin (*purple*)‐positive volume in CTR and NGF‐treated neurons. Images are representatives of at least 50 neurons from 4 to 5 independent neuronal cultures. IPs = Immunoprecipitates. Scale bars: 5 μm.

Finally, we investigated the effect of NGF on the APP interaction with BACE and APP subcellular localization. Both of these events are known to be modulated by APP phosphorylation at T668 (Lee *et al*., 2003; Muresan & Muresan, 2005). For this investigation, we treated SH slices with NGF (100 ng mL^−1^, 1 h), performed an IP analysis with anti‐APP antibody, and probed the samples with specific anti‐TrkA or anti‐BACE1 antibodies. We observed that NGF increases APP binding to TrkA and concomitantly decreases APP binding to BACE1 (Fig. 6A).

To study APP cleavage following NGF treatment, we performed WB for APP, sAPPα, sAPPβ, CTFβ, BACE1 and ADAM17 (Fig. 6B) on untreated and NGF‐treated (100 ng mL^−1^, 1 h) SH slices. We found increased sAPPα (129.4 ± 9.3% of CTR, *P < 0.05) and reduced sAPPβ (50.6 ± 12.2% of CTR, *P < 0.05) and CTFβ (52.5 ± 7.3% of CTR, *P < 0.05) expression upon NGF treatment (Fig. 6C). The levels of full‐length APP (86.2 ± 6.9% of CTR, *P* = 0.12), BACE1 (92.2 ± 18.7% of CTR, *P* = 0.69) and ADAM17 (113.6 ± 5.8% of CTR, *P* = 0.08) were unaffected (Fig. 6C). Moreover, we measured endogenous amyloid‐beta (1‐42) levels in the hippocampus of NGF‐treated SH slices (10 ± 0.1 pg mg^−1^ proteins) and found a significant reduction compared to control (14.5 ± 0.5 pg mg^−1^ proteins, *P < 0.05, Fig. 6D).

To determine whether NGF reduces the interaction between APP and BACE by modulating subcellular APP trafficking, we performed a confocal imaging analysis on NGF‐treated (100 ng mL^−1^; 1 h) septal neurons. Neurons were double‐immunolabelled with several markers for different intracellular compartments, such as giantin for the Golgi, Rab5 for early endosomes, and LAMP1 for late endosomes/lysosomes. Upon NGF stimulation, we found an increase in APP localization at the plasma membrane (Fig. 6E and inset of Fig. 6E, asterisks) and in cell soma, in particular in the Golgi, as quantified with the Imaris 7.6 software (130 ± 9.7% of CTR, **P < 0.01; Fig. 6F; lower magnification in Fig. S3B).

To address APP–BACE interaction in the Golgi, where APP accumulates upon NGF treatment, we performed a triple immunostaining for APP (*red*)/BACE_1_ (*green*)/giantin (*purple*) in septal neurons (Fig. 6G). BACE_1_ immunoreactivity was found to be diffuse and widely distributed throughout the cytoplasm of both unstimulated (CTR) and NGF‐treated (NGF) septal neurons. Moreover, APP and BACE immunofluorescent signals were found to colocalize in the Golgi, although at a low extent (Fig. 6G). Pearson's correlation between APP and BACE within the Golgi has been measured by creating a 3D mask of the giantin‐positive volume, with the help of Volocity 6.0 (PerkinElmer). The 3D rendering of APP (*red*)‐ and BACE (*green*)‐positive voxels within the giantin (*purple*)‐positive volume in CTR and NGF‐treated neurons has been reported in the inset 3D (Fig. 6G). The results of this analysis indicate a moderate correlation of APP and BACE colocalization in the Golgi of CTR neurons (*r* = 0.579 ± 0.084), while poor correlation was found upon NGF treatment (*r* = 0.323 ± 0.049; *P* < 0.05). Of note, BACE localization in the Golgi was not affected by NGF treatment (*P* = 0.314; *r* = 0.363 ± 0.02), as compared to unstimulated neurons (*r* = 0.315 ± 0.05).

APP trafficking to endosomes and lysosomes was also analysed. The amounts of APP localized to the early (Rab5; Fig. S3A) endosomes were comparable (*P* = 0.7) between the CTR (*r* = 0.6 ± 0.2) and NGF‐treated septal neurons (*r* = 0.7 ± 0.1). APP localization to lysosomes (LAMP1; Fig. S3C) was present at a similar extent (*P* = 0.99) in CTR (*r* = 0.5 ± 0.2) and NGF (*r* = 0.5 ± 0.1)‐treated neurons, in our experimental conditions.

Taken together, our data show that NGF treatment favours the APP interaction with TrkA, promotes APP trafficking towards the Golgi, reduces APP exposure to BACE cleavage, and finally results in the reduction of sAPPβ, CTFβ and amyloid‐beta (1‐42) levels in the basal forebrain neurons.

## Discussion

Our study aimed to investigate NGF control of APP phosphorylation and metabolism in the basal forebrain under physiological conditions, and following NGF system perturbation described in AD. NGF is a pleiotropic factor for cholinergic neurons of the basal forebrain, the main target of Alzheimer's disease (AD) pathology. Septal cholinergic neurons have been shown to accumulate intracellular amyloid during aging and AD, thus affecting neuronal activity and cognition in rodents, monkeys and humans (Baker‐Nigh *et al*., 2015; Norvin *et al*., 2015). The data presented in this study indicate that NGF downregulates the phosphorylation of APP at the neuron‐specific residue T668, promotes APP binding with TrkA, and favours APP trafficking to the Golgi, at the expense of BACE interaction and cleavage.

In detail, our findings indicate that ectopic NGF stimulation (Fig. 1A–D) and NGF withdrawal (Fig. 1E–H), control basal APP^pT668^ levels in neurons. Moreover, our results correlated the effect of NGF on the APP^pT668^ level with a specific reduction in JNK(p54) activation. In fact, APP^pT668^ is affected both by pharmacological inhibition of JNKs in SH slices and by genetic ablation of the three JNK isoforms in *JNK*_*1*_, *JNK*_*2*_ and *JNK*_*3*_ KO mice, and of *JNK*_*2/3*_ in particular. Of interest to this study, JNK3 activity has been found to increase by 35% in FAD models, which induces amyloidogenesis and neuronal apoptosis, affects cognition (Yoon *et al*., 2012), and controls APP localization (Muresan & Muresan, 2005).

As expected, also CDK5 inhibitors reduced the APP^pT668^ level in the SH slices (Fig. 2A). However, NGF did not affect the expression levels of CDK5 or its activator p35 (Fig. 2C). Conversely, NGF was able to significantly reduce JNK activation in both the SH slices (Fig 2D–F) and septal neurons (Fig 2G,H). In particular, NGF specifically affected phosphorylation of the JNK(p54) band (Fig. 2F and H). Thus, a crucial role for NGF modulation of JNK(p54) activation may be in APP phosphorylation, metabolism and the related cognition in mammals.

Further analyses focused on the involvement of the SH2‐containing sequence C (ShcC) in these events. ShcC docking to TrkA is an early‐downstream event induced by NGF and causes the PI3K/AKT‐mediated inhibition of JNK (Pelicci *et al*., 2002). Thus, we analysed JNK activation and the APP^pT668^ level in hippocampal extracts from *ShcC* KO mice and found a dramatic rise of JNK phosphorylation (Fig. 3A,C) and a concomitant increase in the APP^pT668^ level compared with age‐matched WT mice (Fig. 3B,C). Furthermore, we found a significant elevation in the total CTF level, and in particular CTFβ (Fig. 3D), while full‐length APP (Fig. 3B) and BACE (Fig. 3E) were unaffected (Fig. 3B,D). Endogenous amyloid‐beta (1‐42) levels were also significantly augmented in *ShcC* null mice, as measured by a rodent‐specific ELISA (Fig. 3D). As a further confirmation of the key role of ShcC in the anti‐amyloidogenic NGF pathway, NGF failed to modulate JNK and APP phosphorylation in SH slices from *ShcC* KO mice (Fig. 3H,I), as well as to reduce CTFβ levels (Fig. 3H). Thus, ShcC is necessary for negative regulation of JNK mediated APP phosphorylation, and the control of the consequent CTFβ and amyloid‐beta generation *in vivo*.

Our data also indicate that TrkA colocalizes with APP in unstimulated septal neurons (CTR; Fig. 4A) and interacts with APP in both hippocampal tissues (Fig. 4C) and SH slices (Fig. 4D). These results are consistent with previous studies demonstrating the APP–TrkA interaction in TrkA‐transfected 293T cells and hippocampal neurons *in vitro* and *in vivo* (Tarr *et al*., 2002; Matrone *et al*., 2011). Moreover, using both confocal imaging (Fig. 4A) and IP analysis (Fig. 4D), we found that the APP association with TrkA increases after NGF treatment. However, TrkA does not colocalize with APP^pT668^ under basal conditions or after NGF treatment (Fig. 4B,C). Indeed, the lack of an APP^pT668^–TrkA interaction was observed in both the control and NGF‐treated SH slices, despite the increase in the amount of total APP bound to TrkA after NGF stimulation (Fig. 4D). These findings lead us to hypothesize that APP and TrkA only form a complex when APP is not phosphorylated at the T668 site and that, in turn, the reduction in the APP^pT668^ level induced by NGF facilitates TrkA binding to APP. Consistent with our model, the interaction between TrkA and APP is almost undetectable in basal forebrain tissues of *ShcC* KO mice (Fig. 4E), in which an elevated level of APP^pT668^ was found (Fig. 3B,C).

Previous studies have shown that transgenic mice lacking the APP–TrkA interaction demonstrate forebrain damage and cognitive deficits (Matrone *et al*., 2012). Based on the effect of APP phosphorylation on APP–TrkA binding, the APP–TrkA interaction was investigated in the AD human brain where elevated APP^pT668^ levels have been reported (Lee *et al*., 2003; Chang *et al*., 2006). An increase in the APP^pT668^ level in patients with AD was also observed in our analysis (Fig. 5A–C). Consistent with our hypothesis, the APP–TrkA interaction was found to be significantly affected (Fig. 5D), and in some cases lost (Fig. 5E), in the AD hippocampus. Interestingly, the AD cerebellum (Fig. 5E), a relatively unaffected tissue in AD (Andersen *et al*., 2012), and the HD hippocampus (Fig. 5D) maintained the APP–TrkA interaction. These data suggest that reduced APP–TrkA binding is a good correlate of AD pathology and not just a general marker of degeneration.

Next, we investigated neuronal APP trafficking and BACE binding/cleavage upon NGF stimulation in primary septal neurons. We found that NGF treatment favoured binding of APP to its specific receptor TrkA at the expense of BACE_1_ (Fig. 6A) and stimulated APP trafficking to the Golgi (arrows, Fig. 6E,F), where APP–BACE colocalization is quite weak in control neurons and further reduced upon NGF treatment of septal neurons (Fig. 6G; inset 3D). These results are in line with recent papers showing that although APP and BACE colocalization is detected in the Golgi, the main neuronal site for APP shedding are the endosomes (Das *et al*., 2013, 2016; Buggia‐Prevot *et al*., 2014; Vassar *et al*., 2014).

Accordingly, NGF treatment promoted the anti‐amyloidogenic processing of APP in the basal forebrain, as demonstrated by the increase in sAPPα and the concomitant decrease in sAPPβ (Fig. 6B,C), CTFβ (Fig. 6B,C) and amyloid‐beta 42 (Fig. 6D) levels in SH slices. Further, these events occurred independently of both BACE and ADAM17 levels (Fig. 6B,C), which were unaffected (Fig. 6C).

Intriguingly, it has been reported that APP is transported using the activated NGF/TrkA‐Shc signalling system in neurons (Heese *et al*., 2004). Therefore, the idea of a NGF‐driven trafficking of APP in septal primary neurons is consistent with previous and recent studies on neuronal APP trafficking and deserves future investigation.

## Conclusions

All together, these data suggest that NGF directly controls the pattern of APP phosphorylation, its trafficking, interaction with and cleavage by BACE, which ultimately reduces CTFβ and amyloid generation in basal forebrain neurons.

Interestingly, APP processing was found to be normal in APP(T668A) mice, bearing a Thr to Ala point mutation (Sano *et al*., 2006), suggesting that APP^pT668^ reduction is not sufficient *per se* to affect APP processing and that instead an active NGF/TrkA–ShcC signalling system is required for promoting APP–TrkA interaction and anti‐amyloidogenic APP processing. Further, the differential binding of APP to its interactors, namely TrkA and BACE, impacting APP trafficking and processing in basal cholinergic neurons is of potential interest for AD pathology, where APP–TrkA interaction was found to be specifically affected.

Therefore, novel strategies aimed at stimulating ShcC signalling and/or promoting TrkA–APP binding should be considered as promising therapeutic tools for early neurodegeneration in AD.

## Experimental procedures

### Reagents and antibodies

Murine NGF was purified from submaxillary glands (Bocchini & Angeletti, 1969). NGF from Xiamen Bioway (Biotech Co., Ltd., Xiamen, Fujian, China) was also used in the study. Roscovitine was purchased from SIGMA (St. Louis, MO, USA; R7772), SP600125 from Calbiochem (San Diego, CA, USA; 420119). Rabbit anti‐TrkA (WB 1:1000, IF 1:100) was a generous gift from Prof. L. Reichardt. The polyclonal anti‐NGF antibody used for NGF neutralization was kindly provided by D. Mercanti (CNR‐IBCN, Rome). The commercial antibodies used are listed in Table 1.

**Table 1:** Antibodies used in the study

Antibody name	Company and code	Working dilution
APP (22c11)	MILLIPORE_(Temecula, CA, USA)_, MAB348	WB 1:1000, IF 1:200
APP C‐ter (Y188)	ABCAM_(Cambridge, UK)_, AB32136	IF 1:100
BACE1	MILLIPORE_(Temecula, CA, USA)_, MAB5308	WB 1:500
BACE1	PIERCE_(Waltham, MA USA)_, PA1‐20215	IF 1:150
ChAT	MILLIPORE_(Temecula, CA, USA)_, AB144P	IF 1:100
APP C‐ter	SIGMA_(St. Louis, MO, USA)_, A8717	WB 1:1000
APP^pT668^	SIGMA_(St. Louis, MO, USA)_, A9103	WB 1:1000, IF 1:100
CDK5	SIGMA_(St. Louis, MO,USA)_, C6118	WB 1:1000
p35	SIGMA_(St. Louis, MO, USA)_, P9489	WB 1:1000
β‐actin‐HRP	SIGMA_(St. Louis, MO, USA)_, A3854	WB 1:1000
sAPPα	IBL INTERNATIONAL_(Hamburg, DE)_, 11088	WB 1:500
sAPPβ	IBL INTERNATIONAL_(Hamburg, DE)_, 18957	WB 1:2000
APP^pT668^	CELL SIGNALING_(Danvers, MA, USA)_, 3823s	WB 1:1000, IF 1:100
pTrkA	CELL SIGNALING_(Danvers, MA, USA)_, 9141	WB 1:1000
SAPK/JNK	CELL SIGNALING_(Danvers, MA, USA)_, 9252s	WB 1:1000
pJNK^T183/Y185^	CELL SIGNALING_(Danvers, MA, USA)_, 9251s	WB 1:1000
Trk	SANTA CRUZ_(Santa Cruz, CA, USA)_, sc7268	WB 1:1000, IF 1:100
Trk	SANTA CRUZ_(Santa Cruz, CA, USA)_, sc11	IF 1:100
Rab5	ABCAM_(Cambridge, UK)_, AB18211	IF 1:100
Giantin	ABCAM_(Cambridge, UK)_, AB24586	IF 1:100
LAMP1	STRESSGEN_(Collegeville, PA, USA)_, ADIVAMEN001	IF 1:100

### SH slices

Young adult C57/Bl6 male mice 3–5 months of age were used for the preparation of SH slices. The animals were sacrificed by cervical dislocation. The SH region was removed and cooled in ice‐cold oxygenated artificial cerebrospinal fluid (aCSF; 126 mm NaCl, 2.5 mm KCl, 1.2 mm Na_2_HPO_4_, 24 mm NaHCO_3_, 2.4 mm CaCl_2_, 1.3 mm MgCl_2_ and 10 mm glucose). Next, 300‐μm‐thick slices were cut using a McILWAIN tissue chopper (Ted Pella, Redding, CA, USA) and incubated in aCSF (1 h) under appropriate oxygenation and pH conditions (95% O_2_, 5% CO_2_). Slices were then exposed to the different treatments, snap frozen and stored at −80 °C until use.

NGF withdrawal experiments were performed by incubation at different time points (3, 6 h) of control SH slices with neutralizing titres of anti‐NGF antibody, as previously described (Matrone *et al*., 2008b).

### Septal neurons primary culture

Septal neurons were prepared from embryonic day 17/18 (E17/18) pregnant Wistar rats or embryonic day 16 (E16) pregnant C57/Bl6 mice, as previously described (Hartikka & Hefti, 1988). They were seeded as follows: 1.5 × 10^6^ cells on poly‐l‐lysine (SIGMA)‐coated plates (BD Falcon, Durham, NC, USA; 353001) for biochemistry analyses and 5 × 10^4^ cells on glass coverslips in 24‐well plates (BD Falcon; 351147) for immunofluorescence analyses. The dissociated cells were plated in Neurobasal medium supplemented with 2% B27 (Invitrogen Inc., Carlsbad, CA, USA) for 10 days (37 °C, 5% CO_2_) and then used for the experiments. At day 10, more than 95% cells showed positive staining for ChAT and around 90% of ChAT‐positive cells were also TrkA positive (data not shown).

### PC12 cell culture

PC12 cells were cultured in RPMI medium containing 10% horse serum and 5% FBS. NGF deprivation was performed as previously described (Matrone *et al*., 2008b). PC12 cells were incubated with NGF (100 ng mL^−1^) for 10 days until fully differentiated and then used as a control (CTR) or kept in serum‐free and NGF‐free medium for 30 min or 3 h (NGF‐dep 30′; NGF‐dep 3 h). Plates were frozen at −20 °C until use.

### Rodent strains

All animals were handled in compliance with the national (D.Lgs26/2014) and international guidelines for animal welfare (2010/63/EU). All efforts were made to minimize the number of animals used and suffering. C57/Bl6J mice and Wistar rats (HARLAN Laboratories Ltd., Füllinsdorf, Switzerland) were housed at the IBCN‐CNR (Italy) animal facility. Brain tissues from male *ShcC* KO mice (Sakai *et al*., 2000), maintained in a C57/Bl6J background, were collected from mice housed at the Animal Facility of the ‘University of Siena’ (Italy). The *JNK*1‐3 KO mouse hippocampal tissues were provided by M.F. (Nisticò *et al*., 2015). Age‐matched C57/Bl6J mice and/or tissues were used as controls in this study.

### Human brain samples

Brain tissue samples from human subjects were provided by the Regional Neurogenetic Center (CRN, ASP CZ) of Lamezia Terme, Italy. All brain donors or their legal tutors gave written informed consent during their lifetime, and the protocol was approved by the local ethics committee. The study was conducted with brain samples from 3 unrelated patients affected by familial AD (FAD) and bearing the Met146Leu mutation of the Presenilin1 gene. The mean age of death of the patients was 48 ± 8.2 years, and they were at the terminal stage of dementia (stage 5, according to the Clinical Dementia Rating scale). For all patients, the Braak staging for AD pathology (AD‐related neurofibrillary pathology) was VI. Brain tissues from three human subjects (CTR; 60 ± 7 years of age) without neurological diseases were used as control. Samples from three subjects affected by other neurological diseases (OND) were also analysed in this study. Specifically, the three OND subjects were affected by spinocerebellar ataxia 17 (SCA17), Huntington's disease (HD), and the third subject died from accidental intracerebral bleeding in the left brain hemisphere (the sample was taken from the counter‐lateral right brain hemisphere). Histopathological lesions suggestive of AD (neurofibrillary pathology, deposition of Aβ protein) were excluded. In details, tissues were sampled from hippocampal gyrus (CA1, CA2, CA3 and dentate gyrus, excluding parahippocampal gyrus and subiculum) and from cerebellar hemispheres. The brain samples were collected at 4–36 h postmortem and stored at −80 °C until use.

### WB analysis

Brain slices, tissues samples and cultured neurons were digested in a RIPA buffer with phosphatase and protease inhibitors (Pierce) and centrifuged (10,000 rpm, 20′). The supernatants were collected, and the amount of total protein was determined (Quick Start Bradford Dye Reagent, Biorad, Hercules, CA, USA). Each sample (40 μg) was separated by SDS–PAGE in 4–12% Bis‐Tris gels (Invitrogen), transferred to nitrocellulose membranes (0.45 μm, GE healthcare, Little Chalfont, UK) and incubated for 1 h at room temperature with 5% nonfat dry milk in TBS‐T (10 mm Tris, pH 7.5, 100 mm NaCl and 0.1% Tween‐20). The overnight incubation with primary antibody (4 °C) was followed by incubation with the appropriate HRP secondary antibody (1:3000, Pierce, 1 h, RT) and the ECL method (GE Healthcare). The films were digitalized using a professional scanner (HP) and quantified using image 1.63 software (NIH). Omission of the primary antibody was routinely performed as control for antibody specificity (not shown).

### Immunoprecipitation

A total of 1* *mg of protein extract was added to Dynabeads‐Protein G and eluted with 0.1 m citrate buffer, pH 2.3, according to the manufacturer's instructions (Invitrogen). IP of APP was performed using rabbit anti‐APP C‐terminal antibody (Sigma; 10 μg/50 μL beads). IP of TrkA was performed using specific anti‐TrkA antibody (4 μL/50 beads). There were no specific bands detected by WB, when the same amount of normal rabbit IgG (Santa Cruz, sc‐2027) was used as isotypic control for the IP (Fig. S1E).

### Immunofluorescence and confocal imaging

Primary septal cell cultures were fixed for 20 min in PBS containing 4% paraformaldehyde (PFA), permeabilized and quenched. Statistical analysis was performed and representative images were chosen among 50 primary neurons from multiple assays.

*ShcC* KO (*n* = 3) and age‐matched control mice (*n* = 3) were anesthetized with avertin (500 mg kg^−1^, i.p.; Sigma‐Aldrich) and perfused transcardially with PBS, pH 7.4, followed by 4% PFA in PBS. Then, the brains were collected, postfixed overnight (4% PFA, 4 °C) and cryoprotected in 30% (w/v) sucrose in PBS 0.1 m (4 °C) until they sank to the bottom, as previously described (Müller *et al*., 2012). Coronal brain sections (25 μm thick) were cut on a cryostat (Leica Microsystems) and kept floating in PBS at 4 °C until use. Sections spanning from bregma 0.74 mm to 1.34 mm (medial septum) and from bregma −1.34 to −2.3 mm (hippocampus) were used for this analysis (Paxinos & Franklin, 2001).

Coronal brain sections or fixed primary neurons were incubated with ammonium chloride (50 mm, RT) for 30 min, to quench autofluorescence and then blocked with 10% normal donkey serum (code: 01700121, Jackson Lab, Bar Harbor, ME, USA). The overnight incubation (4 °C) with primary antibody was followed by incubation with the appropriate combination of secondary antibodies. In particular, donkey anti‐mouse‐546 and donkey anti‐rabbit‐488, or donkey anti‐mouse‐488 and donkey anti‐rabbit‐546 were used for double immunofluorescence; donkey anti‐mouse‐647, donkey anti‐goat‐546 and donkey anti‐rabbit‐488 were used for triple immunostaining (Life Technologies, 1 h, RT, Carlsbad, CA, USA). Finally, DAPI (Life Technologies) was used for counterstaining and Prolong Gold (Life Technologies) for mounting on Superfrost Plus slides. Confocal microscopy was performed with the laser scanning confocal microscope TCS SP5 (Leica Microsystems, Mannheim, Germany) using a 40X (NA = 1.25) and a 63X (NA = 1.4) oil‐immersion lens. An UV diode laser operating at 405 nm, an Argon laser at 488 nm and HeNe lasers at 543 or 633 nm were used as excitation sources. Confocal Z‐stacks were collected at 5X zoom at 0.29 mm intervals in a 4 μm total optical depth. Images for direct comparison were collected using the same parameters and were analysed with the help of imaris 7.6 (Bitplane, Zurich, Switzerland) or volocity 6.0 (PerkinElmer, Waltham, MA, USA) softwares. Pearson's correlation coefficient (*r*) was used for correlation analysis, with an r value between +0.5 and 1.0 indicating a high strength of correlation. Omission of the primary antibody was routinely performed as control for antibody specificity (not shown). The specificity of the antibody against BACE1 was tested by pre‐incubation of the primary antibody with the BACE peptide (PEP 0077, ThermoFisher, Waltham, MA, USA), resulting in no staining (not shown).

### Amyloid‐beta (1‐42) ELISA

To measure endogenous amyloid‐beta (1‐42) levels, a mouse‐/rat‐specific and high‐sensitive enzyme immunoassay kit was used, according to the manufacturer's protocol (code: re45721; IBL Int.).

### Statistical analysis

All experiments using SH slices and primary neurons were performed at least four times independently, each in triplicate. Tissues from four *ShcC* KO and four control mice were analysed. The graphs were generated using prism (GraphPad Software, Inc., San Diego, CA, USA), and the data are presented as the mean ± standard error. Student's *t*‐test or anova followed by the Tukey–Kramer *post hoc* was used to analyse the data depending on the number of variables and groups (Statview‐SAS, Cary, NC, USA). A *P* value ≤ 0.05 was considered statistically significant.

## Author contributions

VT designed and performed the experiments, analysed the data and wrote the manuscript; VS performed the experiments and contributed to M&M; GB performed confocal microscopy; TC prepared the neuronal cultures and slices; PP collaborated with the *ShcC* KO mice; ACB and RM enrolled the patients with AD; CC prepared the AD brain samples; NC contributed to the interaction analysis and manuscript writing; M.F and R.N provided JNK KO mouse tissues and expertise; and PC supervised the study and wrote the manuscript.

## Funding

This study was supported by FIRB funding RBAP10L8TY_004 to PC.

## Conflict of interest

The authors declare no conflict of interest.

## Supporting information


**Fig. S1** NGF reduces pAPP (A) and pJNK (B) levels in rat primary septal neurons (E18, DIV10).

**Fig. S2** Low magnification confocal images of APP, APP^pT668^ and TrkA localization in septal neurons.

**Fig. S3** Low magnification confocal images of APP trafficking under NGF treatment showing that NGF (100 ng mL^−1^, 1 h) affects Golgi accumulation of APP but not endosomal or lysosomal APP localization in primary septal neurons (E17, DIV10).
