# Supplementary figures and images for: NGF controls APP cleavage by downregulating APP phosphorylation at Thr668: relevance for Alzheimer's disease
Source: Aging Cell. 2016 Apr 13;15(4):661–72. doi: 10.1111/acel.12473 (PMC4933663; doi:10.1111/acel.12473)

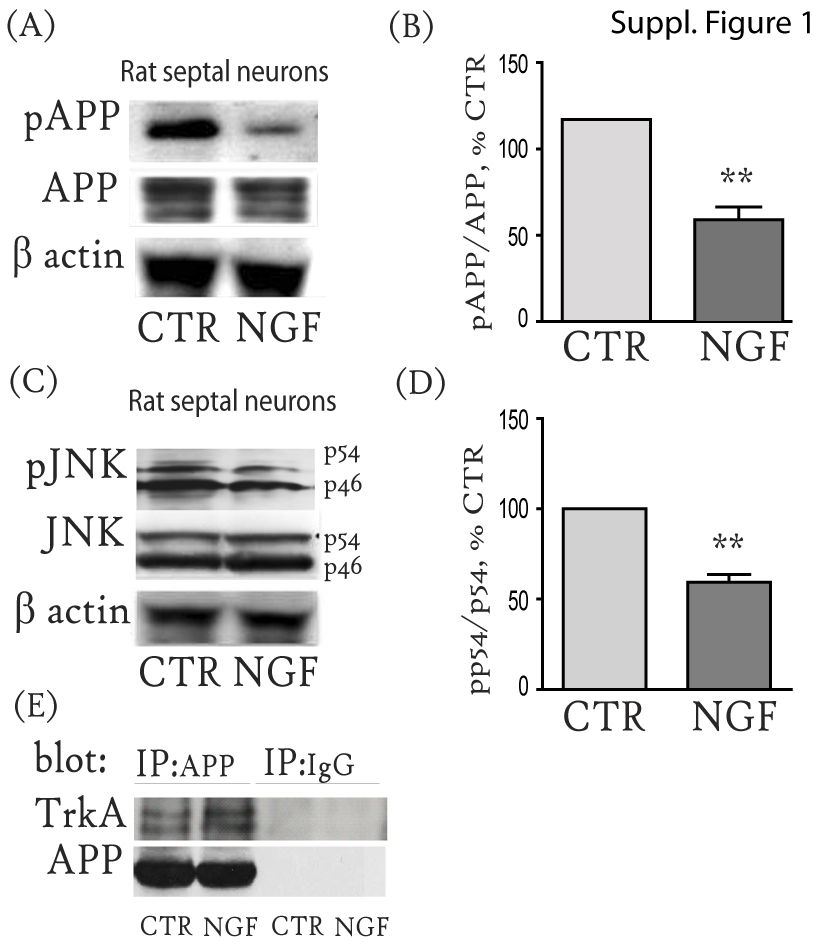

Supplement: Supplementary file 1 — Fig. S1 NGF reduces pAPP (A) and pJNK (B) levels in rat primary septal neurons (E18, DIV10). [file ACEL-15-661-s001.tif]

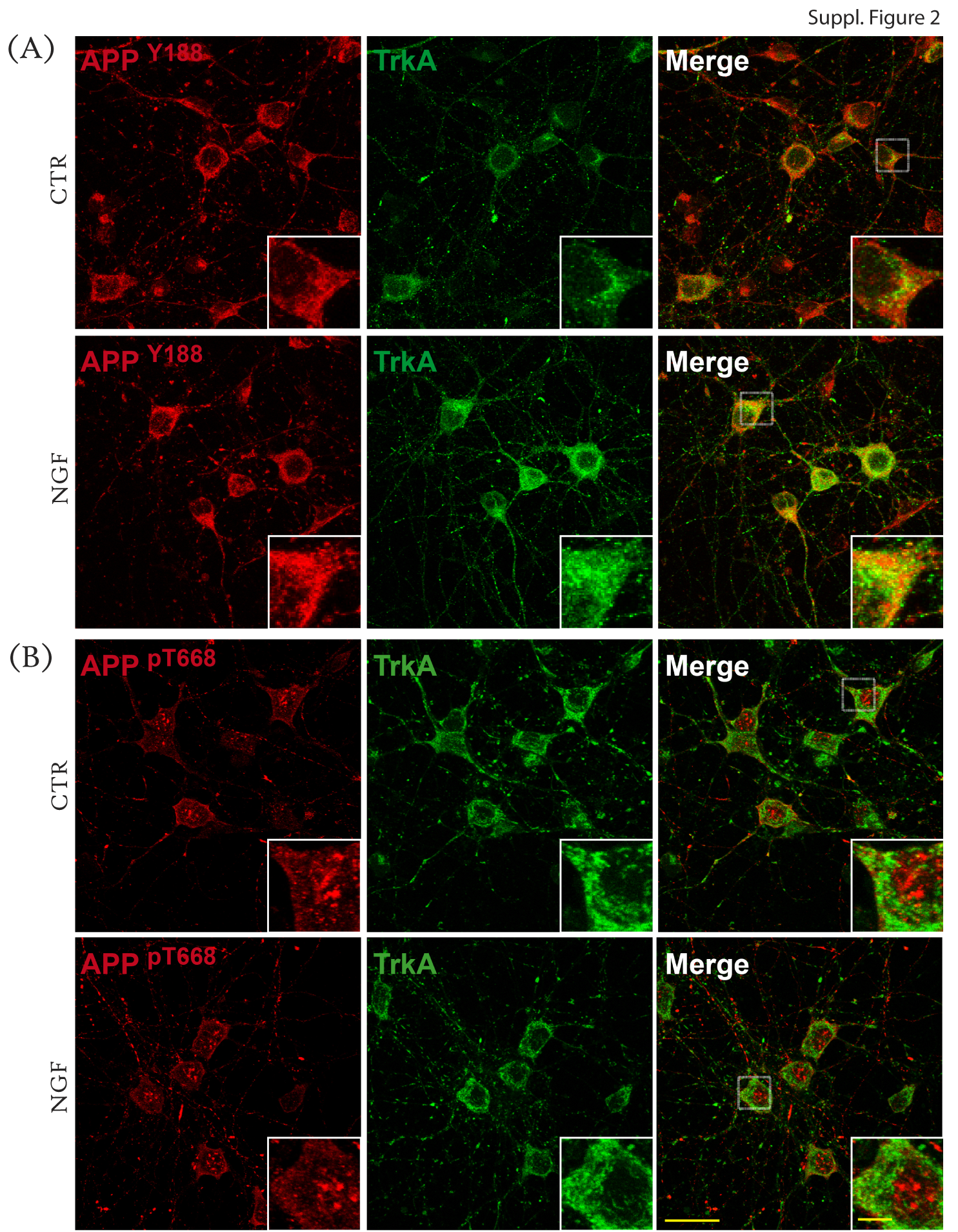

Supplement: Supplementary file 2 — Fig. S2 Low magnification confocal images of APP, APPpT668 and TrkA localization in septal neurons. [file ACEL-15-661-s002.tif]

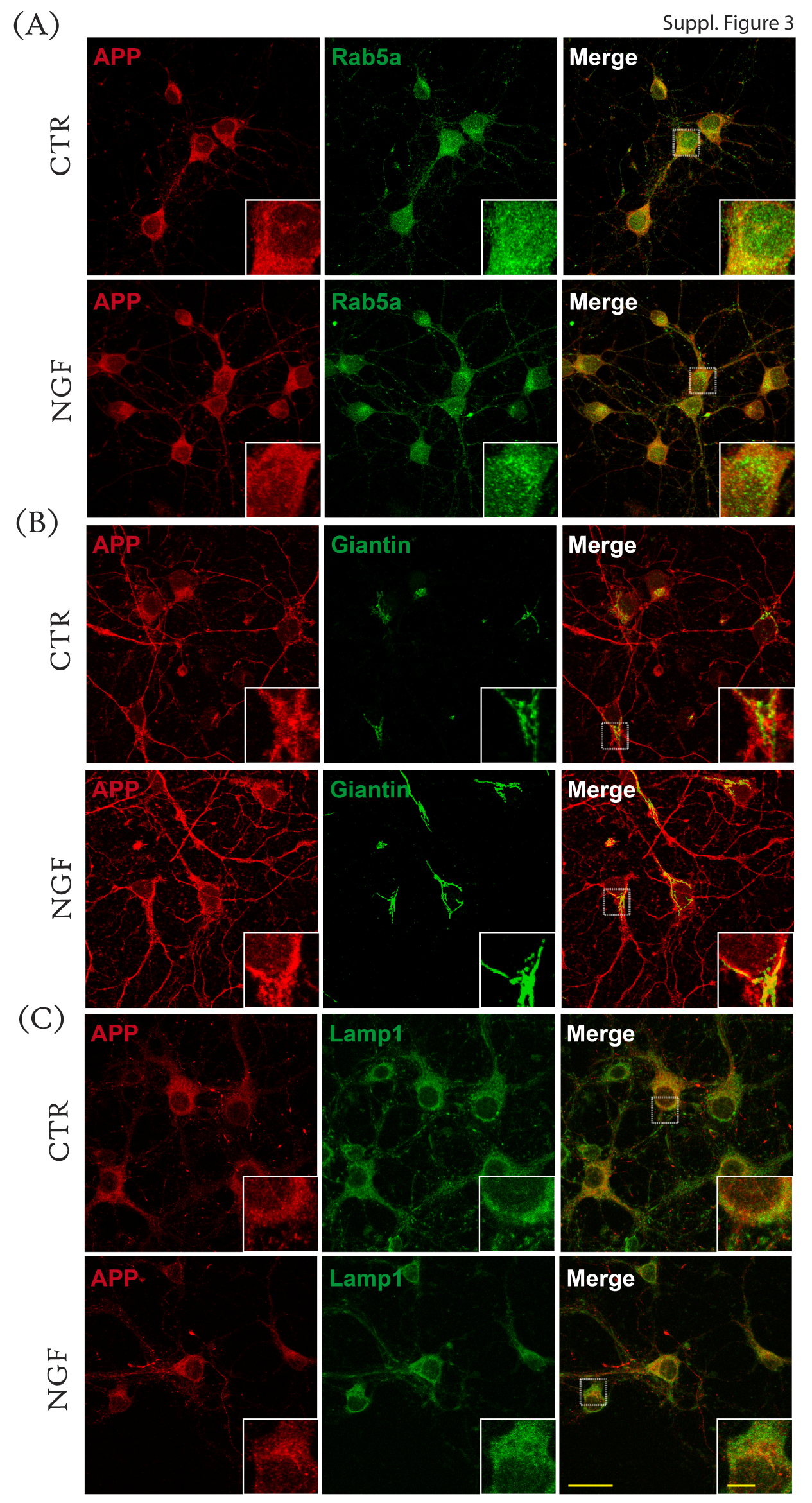

Supplement: Supplementary file 3 — Fig. S3 Low magnification confocal images of APP trafficking under NGF treatment showing that NGF (100 ng mL−1, 1 h) affects Golgi accumulation of APP but not endosomal or lysosomal APP localization in primary septal neurons (E17, DIV10). [file ACEL-15-661-s003.tif]
